# Supplementary material for: HCV Infection and Liver Cirrhosis Are Associated with a Less-Favorable Serum Cholesteryl Ester Profile Which Improves through the Successful Treatment of HCV
Source: Biomedicines. 2022 Dec 6;10(12):3152. doi: 10.3390/biomedicines10123152 (PMC9775323; doi:10.3390/biomedicines10123152)
Supplement: Supplementary file 1 [file biomedicines-10-03152-s001.zip › biomedicines-2034440-supplementary.pdf]

HCV infection and liver cirrhosis are associated with a less-favorable serum cholesteryl ester profile which improves through successful treatment of HCV

Kilian Weigand [1, 2], Georg Peschel [1,3], Jonathan Grimm [1], Martina Müller [1], Marcus Höring [4], Sabrina Krautbauer [4], Gerhard Liebisch [4] and Christa Buechler[*, 1]

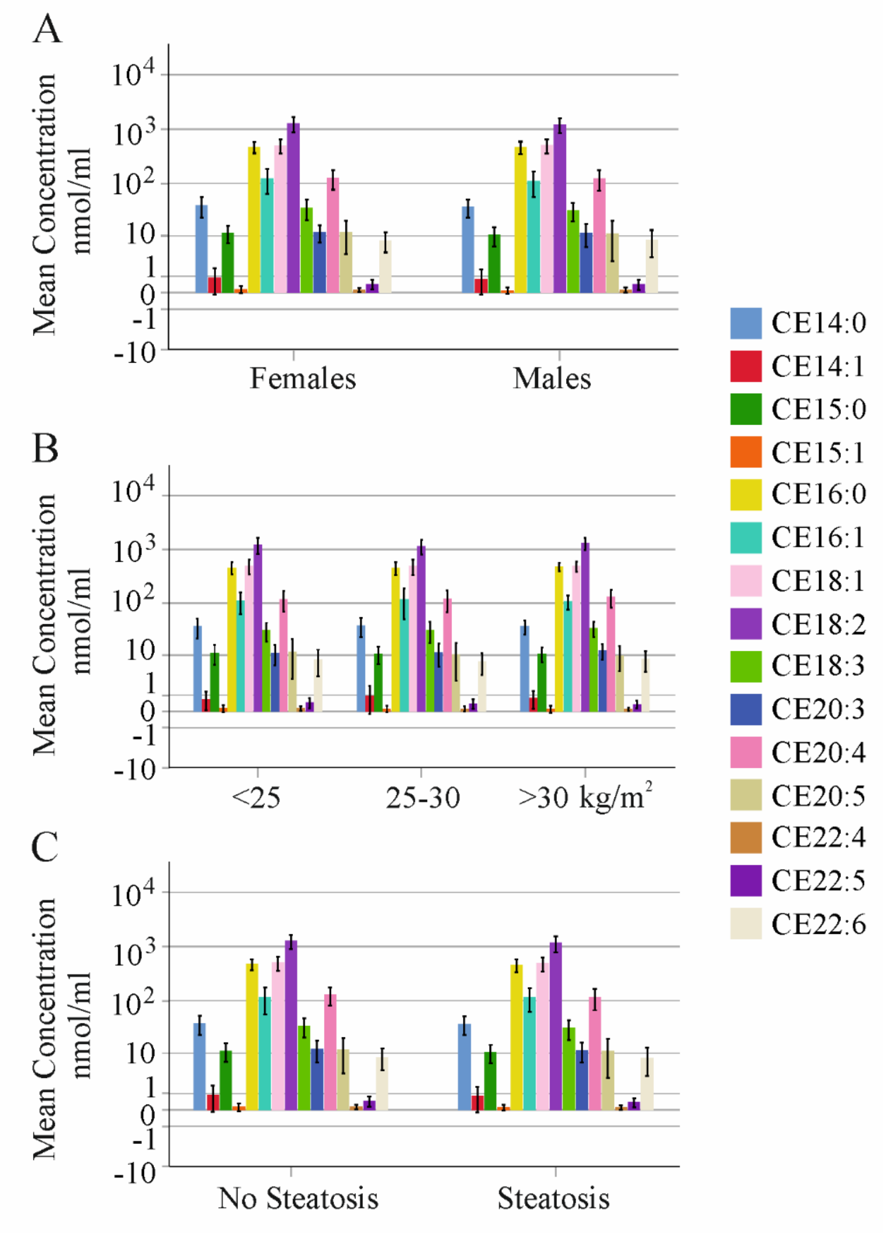

Supplementary Fig. S1. Serum cholesteryl ester (CE) species in relation to gender, body mass index (BMI) and steatosis in patients with chronic HCV. **A** CE species in female and male patients. **B** CE species stratified for BMI. **C** CE species in patients with and without liver steatosis.

Supplementary Table S1. Spearman correlation coefficients of the association of CE species and FC with age and BMI in the whole cohort before therapy. None of these correlations was significant.

CE	14:0	14:1	15:0	15:1	16:0	16:1	18:1	18:2
**Age**	0.126	0.070	0.115	0.063	-0.043	0.072	-0.069	-0.135
**BMI**	0.008	0.046	-0.019	-0.132	0.076	0.024	-0.001	0.070

CE	18:3	20:3	20:4	20:5	22:4	22:5	22:6	FC
**Age**	-0.107	-0.126	-0.196	-0.153	-0.088	-0.177	-0.163	0.031
**BMI**	0.079	0.096	0.086	-0.083	-0.094	-0.115	0.045	0.122

Supplementary Table S2. Concentration of FC (nmol/ml) in relation to gender, BMI, liver steatosis and diabetes of patients with HCV before therapy start.

	Median	Minimum	Maximum	P-value
		**Gender**		
**Females**	1018	588	1738	0.565
**Males**	1077	218	1747	
		**BMI**		
**< 25 kg/m^2**	1004	560	1747	0.305
**25-30 kg/m^2**	1025	218	1610	
**> 30 kg/m^2**	1102	654	1452	
		**Steatosis**		
**No**	1044	562	1747	0.499
**Yes**	1072	218	1610	
		**Diabetes**		
**No**	1044	218	1747	0.307
**Yes**	1105	689	1610	

Supplementary Table S3. Spearman correlation coefficients of the association of FC and CE species with viral load before therapy. None of these associations was significant.

CE	No Cirrhosis	Cirrhosis
**14:0**	0.132	0.289
**14:1**	0.104	0.222
**15:0**	0.064	0.128
**15:1**	0.103	0.144
**16:0**	0.044	-0.082
**16:1**	0.083	-0.041
**18:1**	0.175	-0.071
**18:2**	-0.032	-0.116
**18:3**	0.081	0.059
**20:3**	0.119	0.083
**20:4**	-0.117	-0.032
**20:5**	0.032	0.158
**22:4**	-0.107	-0.151
**22:5**	-0.053	0.252
**22:6**	-0.045	0.009
**FC**	0.090	-0.139
